# Temporal Dynamics of Sensitivity to Second-Order Facial Relations: Evidence from a Rapid ERP Adaptation Paradigm

**DOI:** 10.3390/bs16081301

**Published:** 2026-07-31

**Authors:** Xiaoli Ma, Danyang Cui, Xiaohua Cao

**Affiliations:** Department of Psychology, Ningbo University, Ningbo 315211, China; maxiaoli@nbu.edu.cn (X.M.); 2411030203@nbu.edu.cn (D.C.)

**Keywords:** second-order relational processing, face perception, adaptation, configural processing, ERP

## Abstract

Second-order facial relations—the metric spacing among internal facial features—contribute to face individuation, but the temporal stages at which the brain differentiates repeatedly from changed relational information remain unclear. Using a rapid-adaptation EEG paradigm, seventy adults viewed S1–S2 face pairs derived from the same source identity, in which the spacing of the eyes and mouth was either repeated or changed while first-order facial layout was preserved. ERPs to S2 were analysed in the P1, N170, P2, and N250 time windows and complemented by cluster-based permutation tests. Repeated versus changed spacing did not reliably modulate the P1 or N170. Instead, a sustained posterior condition difference emerged after the N170 and extended across the conventionally defined P2 and N250 time ranges, with repeated spacing eliciting a more positive-going response than changed spacing across this interval. Cluster-based analyses confirmed that this difference extended across contiguous post-N170 time points and posterior electrodes. Under the present rapid-adaptation conditions, neural differentiation of repeated versus changed second-order relations was therefore most reliably expressed in later P2 and N250 activity. These findings do not imply that second-order information is absent from earlier processing stages, but identify a sustained post-N170 interval in which repeated and changed second-order relations were reliably differentiated.

## 1. Introduction

To recognize an individual across changes in viewpoint, lighting, and expression, the visual system must extract information that distinguishes one face from many visually similar alternatives. A central proposal in the face-processing literature is that this individuation depends not only on local facial features, but also on configural processing—the encoding of spatial relations among facial features (e.g., Farah et al., 1998; Maurer et al., 2002; Poltoratski et al., 2021; Rossion, 2013). Within this broader construct, Maurer et al. (2002) distinguished three forms of configural processing: first-order relations, which refer to the basic spatial layout that defines a stimulus as face-like, such as two eyes above a nose and mouth; holistic processing, which refers to the integration of facial parts into a unified perceptual whole; and second-order relations, which refer to metric distances among facial features, such as interocular spacing or the relative position of the eyes and mouth. Behavioral studies have provided substantial evidence for the role of second-order relations in face recognition (e.g., Cattaneo et al., 2014; Freire et al., 2000; Itz et al., 2018; Leder & Bruce, 2000; Rotshtein et al., 2007; J. W. Tanaka & Farah, 1991). For example, subtle changes in the spacing among the eyes and mouth can impair recognition, and such sensitivity is reduced when faces are inverted, suggesting that second-order relational processing contributes to the well-known face inversion effect (Freire et al., 2000; Leder & Bruce, 2000; Taubert et al., 2011). However, behavioral measures alone provide limited information about when, during the unfolding of visual processing, the brain becomes sensitive to second-order relational changes. The temporal dynamics of second-order relational processing therefore remain a critical unresolved issue.

Event-related brain potential (ERP) methods, with their millisecond temporal resolution, provide an ideal tool for parsing the successive stages of face processing, and several components have been linked to distinct aspects of face perception (e.g., Gosling & Eimer, 2011; Nemrodov et al., 2016; Schweinberger & Neumann, 2016; H. Tanaka & Jiang, 2024). The P1 component, peaking approximately 80–130 ms over posterior occipital sites, indexes early sensory-perceptual processing that is sensitive to both low-level visual properties and attentional allocation (e.g., Hillyard et al., 1998; Martínez et al., 2001; Mishra et al., 2012). Although P1 responses can be modulated by faces, these effects are often difficult to separate from low-level image differences and are not typically taken as evidence for face-selective configural or identity processing (e.g., Rossion & Jacques, 2011; Schindler et al., 2021). The N170 component, a negative-going ERP response peaking approximately 130–200 ms over occipito-temporal scalp sites, is the most extensively studied face-sensitive ERP component (e.g., Bentin et al., 1996; Eimer, 2011; Rossion & Jacques, 2008, 2011). It is typically larger for faces than for many non-face object categories, often shows a right-hemisphere bias, and is reliably delayed and enhanced by face inversion (Alzueta et al., 2021; Anaki et al., 2007; Jacques et al., 2007). These properties have led to the view that the N170 reflects an early stage of face-sensitive structural encoding and configural face processing (e.g., Eimer et al., 2011; Rossion & Jacques, 2011). However, configural processing is not a unitary construct (Maurer et al., 2002), and evidence that the N170 reliably differentiates fine-grained metric spacing among internal facial features remains mixed. Several studies have found little or no P1 or N170 modulation when internal-feature spacing was changed while first-order facial layout was preserved (Halit et al., 2000; Lv et al., 2015; Mercure et al., 2008), whereas others have reported N170 effects under familiarisation or adaptation conditions (Scott & Nelson, 2006; Vakli et al., 2014; Zimmer & Kovács, 2011). Taken together, these findings suggest that second-order relational information can influence the N170 under some conditions, but that such modulation is not a consistent neural signature of fine-grained metric-spacing sensitivity.

Later ERP components provide theoretically distinct candidate windows for second-order relational processing. The occipitotemporal P2, peaking approximately 200–250 ms, has been linked to the processing and evaluation of a face’s second-order spatial configuration (Burkhardt et al., 2010; Kaufmann & Schweinberger, 2012; Schulz et al., 2012; Wang et al., 2020; Wang et al., 2022; Wuttke & Schweinberger, 2019). Evidence from photorealistic spatial caricaturing and anti-caricaturing indicates that P2 amplitude varies with the prototypicality of metric inter-feature layout, suggesting sensitivity to how closely a face conforms to, or deviates from, a current facial prototype (Kaufmann & Schweinberger, 2012; Schweinberger & Neumann, 2016). Similarly, compressing or expanding the inner features of a face—a manipulation that directly targets second-order relational distances—modulates P2 amplitude (Burkhardt et al., 2010). The subsequent N250, a posterior occipitotemporal negativity typically peaking around 230–330 ms, has been associated with face repetition, familiarity, and the acquisition or activation of individual-face representations (Schweinberger et al., 2002; J. W. Tanaka et al., 2006; H. Tanaka & Jiang, 2024; Wuttke & Schweinberger, 2019). The P2 may therefore provide a candidate index of spatial-relational evaluation, whereas the N250 provides a later window onto repetition-sensitive and representation-related processing. If second-order relational continuity contributes to the short-term processing of a face, repeating or changing internal-feature spacing may modulate the P2, the N250, or both, even when earlier effects are weak or absent. A rapid adaptation paradigm provides a direct way to trace this temporal progression. In this paradigm, two face stimuli are presented in rapid succession—an adaptor face (S1) followed by a test face (S2)—and neural responses to S2 are compared according to whether a particular facial property is repeated or changed relative to S1 (Eimer et al., 2010; Feuerriegel et al., 2015; Tian et al., 2015). Repetition-related effects can be expressed as either amplitude suppression or enhancement, depending on the processing stage, stimulus property, and experimental context (Benda, 2021; Brands et al., 2024; Henson et al., 2003; Larsson & Smith, 2012). This paradigm is therefore useful not only for determining when the visual system becomes sensitive to repeated versus changed second-order relations, but also for examining whether the direction of this sensitivity differs across successive processing stages.

The present study applied this approach by recording ERPs from healthy young adults during a rapid S1–S2 adaptation task. S1 and S2 were derived from the same source identity, while the metric spacing between the eyes and mouth was either repeated across the pair (Adapt condition) or changed (Nonadapt condition), with first-order facial layout maintained. Analyses of the P1 and N170 assessed whether relational continuity influenced early sensory-perceptual and face-structural processing, whereas analyses of the P2 and N250 examined later spatial-relational and repetition-sensitive stages. Cluster-based permutation tests complemented the component analyses by characterising the broader spatiotemporal distribution of the Adapt–Nonadapt effect without restricting inference to predefined electrodes or time windows.

The primary aim was to characterise the temporal progression of neural sensitivity to repeated versus changed second-order facial relations across the P1, N170, P2, and N250 time ranges. Based on previous evidence, we expected little or inconsistent modulation at the P1 and N170, but more reliable differentiation in the P2 and/or N250 ranges.

## 2. Materials and Methods

### 2.1. Participants

A total of 76 healthy young adults were initially recruited from Ningbo University. For three participants, however, EEG data were unavailable because of equipment failure; these participants were therefore excluded from the ERP analyses. Another three participants were excluded because of insufficient task engagement: two participants had sensitivity scores (d′) more than three standard deviations below the sample mean, and one participant showed an extremely low target hit rate (<10%). After these exclusions, the final sample comprised 70 participants with available EEG data who met the behavioural-engagement and ERP inclusion criteria (40 females; age range = 18–26 years, *M* = 20.04, *SD* = 2.24). For context, the present sample (N = 70) was larger than those analysed in several related face-ERP adaptation studies (Eimer et al., 2010; Feuerriegel et al., 2015; Vakli et al., 2014). A sensitivity analysis conducted using G*Power 3.1 indicated that, with a two-tailed α level of 0.05 and 80% target power, the minimum detectable effect for a single paired within-participant contrast was approximately dz = 0.34. This benchmark is most directly interpretable for a single paired contrast and does not provide a design-wide estimate of sensitivity for all ANOVA effects or the spatiotemporal cluster-based permutation analyses. All participants were native Chinese speakers. They reported normal or corrected-to-normal vision and no history of neurological or psychiatric disorders. All participants were right-handed, as assessed by self-report based on preferred hand use across multiple daily activities (writing, drawing, brushing teeth, and using chopsticks). Written informed consent was obtained from all participants prior to testing. Participants received 50 RMB for their participation. The study was approved by the Ethics Committee of the Department of Psychology, Ningbo University and was conducted in accordance with the Declaration of Helsinki.

### 2.2. Stimuli

The stimulus set comprised 16 unfamiliar face identities selected from a larger laboratory stimulus pool. The identities were purposely selected to create a balanced Ethnicity × Sex design, with four identities in each category: four Asian women, four Asian men, four Caucasian women, and four Caucasian men. Eligible images were required to depict a frontal pose and neutral expression, to have adequate image quality, and to contain no conspicuous external identifying features. The selection was therefore criterion-based rather than random, and all 16 selected identities were included in the main experiment. All stimuli were normalized for size and position and subtended approximately 4.7° × 6.3° of visual angle at a viewing distance of 70 cm.

**Figure 1:**
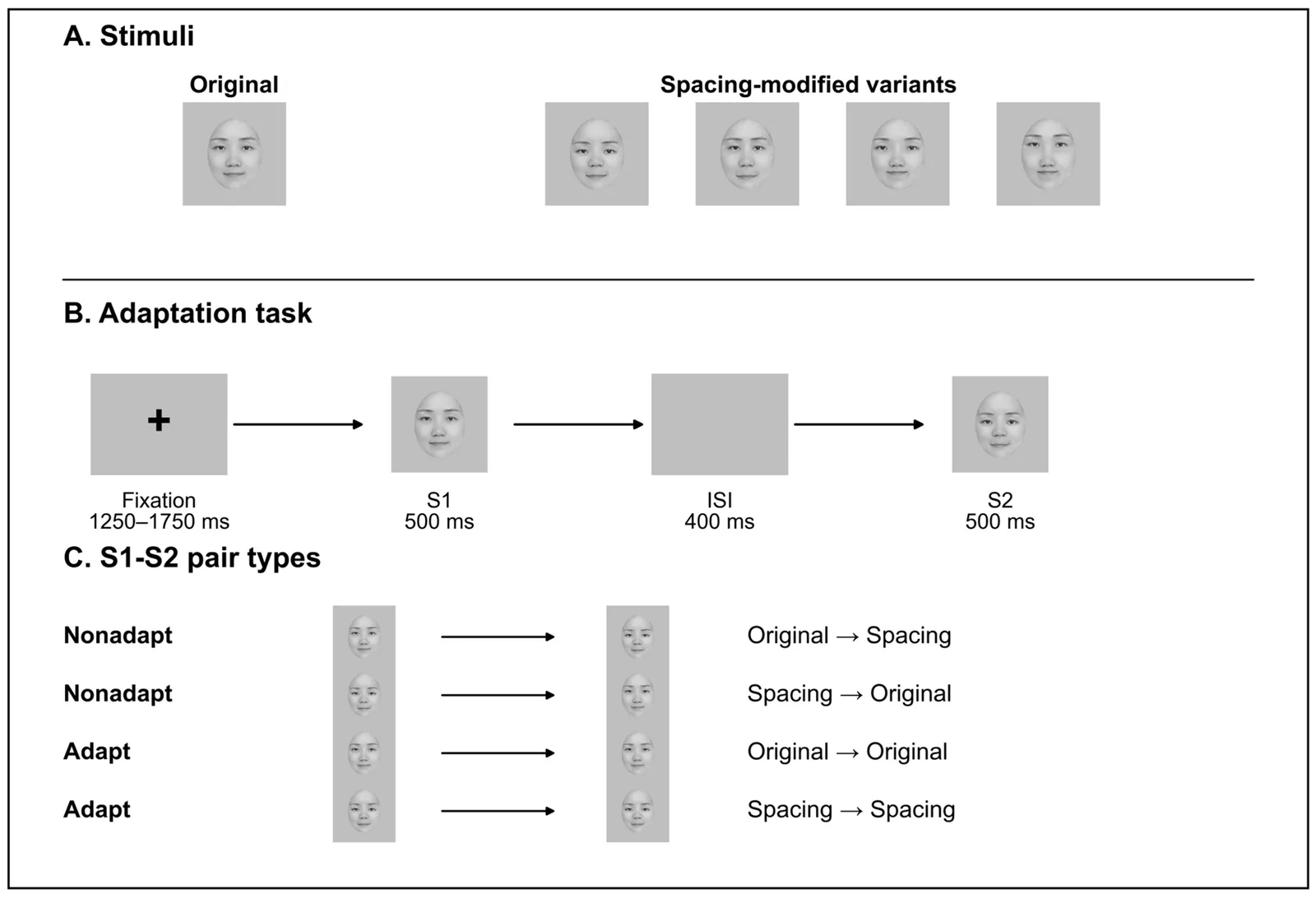
Stimuli and S1–S2 adaptation paradigm. (**A**) An original face and four spacing-modified variants created by shifting the eyes and mouth while preserving source identity and first-order layout. (**B**) Trial sequence: fixation (ITI, 1250–1750 ms), S1 (500 ms), a blank interstimulus interval (ISI; 400 ms), and S2 (500 ms). (**C**) S1–S2 pair types. In Nonadapt trials, S1 and S2 were derived from the same identity but differed in internal feature spacing; in Adapt trials, the same image was repeated across S1 and S2. The arrows in Panel B indicate temporal sequence of events of a trial, whereas those in Panel C indicate the direction of S1–S2 pairing.

To manipulate second-order relational information, four altered configurations were constructed for each identity in addition to the original face. These modifications changed the metric distances among the eyes and mouth while leaving the first-order face structure intact. The four altered configurations involved (Mondloch et al., 2002): (a) downward displacement of the eyes by 4 mm together with downward displacement of the mouth by 2 mm; (b) a 4-mm reduction in interocular spacing together with a 2-mm downward displacement of the mouth; (c) a 4-mm increase in interocular spacing together with a 2-mm upward displacement of the mouth; and (d) upward displacement of the eyes by 4 mm together with upward displacement of the mouth by 2 mm. These modifications were made using Adobe Photoshop CS6. In total, 80 face images were included in the stimulus set: the original and four spacing-modified versions of each of the 16 identities (see Figure 1).

### 2.3. Procedure

Participants were tested individually in an electrically shielded EEG laboratory. They were seated approximately 70 cm from the monitor and were instructed to remain still and maintain fixation during stimulus presentation. The experiment was implemented in E-Prime 3.0 (Psychology Software Tools, Pittsburgh, PA, USA). All face stimuli were displayed centrally against a light-gray background.

The rapid-adaptation task comprised 144 trials, arranged into two blocks of 72 trials. Face sex was held constant within each block, such that one block presented eight male identities and the other presented eight female identities; the order of the two blocks was counterbalanced across participants. As illustrated in Figure 1B, each trial began with a central fixation cross presented for a randomly jittered duration of 1250–1750 ms, followed by an adaptor face (S1) for 500 ms. After a blank interstimulus interval of 400 ms, a test face (S2) was presented for 500 ms. S1 and S2 were always derived from the same source identity. In the Adapt condition, the two sequential faces had identical internal-feature configurations, consisting of either an original face followed by the same original face or a spacing-modified face followed by the same modified face. In the Nonadapt condition, the internal-feature spacing differed between S1 and S2; these trials included both original-to-modified and modified-to-original transitions (Figure 1C). To ensure sustained attention to the face stimuli without explicitly directing participants to the second-order manipulation, an ethnicity-change detection task was used. Participants were instructed to press the space bar with their right hand whenever the ethnicity of the faces on the current trial differed from that on the immediately preceding trial. These target trials accounted for 14 out of 144 trials (9.7%) in total. Because the ethnicity-change task required comparison with the immediately preceding trial, the first trial of each block was excluded from the ERP analyses. After these task-based exclusions, the nominal ERP trial pool comprised 128 nontarget trials before EEG artifact rejection, equally divided between the Adapt and Nonadapt conditions (64 trials each). Each of the 16 identities contributed eight analysable trials: four Adapt trials, including two original-original repetitions and two modified-modified repetitions, and four Nonadapt trials, including two original-to-modified and two modified-to-original transitions. Thus, presentation frequency and Pair Type allocation were balanced across the 16 identities.

Participants completed a short practice session before the EEG task to ensure that they understood the response rule. Brief rest periods were provided after every 18 trials, with an additional longer break between the two experimental blocks.

Following the EEG session, participants completed the Cambridge Face Memory Test-Chinese version (Duchaine & Nakayama, 2006; McKone et al., 2017); details and exploratory ERP-behaviour association analyses are reported in Supplementary Analysis S1.

### 2.4. EEG Recording and Preprocessing

Continuous EEG was acquired from 32 Ag/AgCl scalp electrodes mounted in an elastic cap (Easycap GmbH, Herrsching, Germany) and positioned according to the extended International 10–20 system. The EEG was recorded using BrainAmp amplifiers (Brain Products GmbH, Gilching, Germany) at a sampling rate of 1000 Hz. Cz served as the online reference and AFz as the ground electrode. Electrode impedances were maintained below 10 kΩ throughout recording.

Offline processing was performed in MNE-Python (version 1.11.0; Gramfort et al., 2014). Channels showing persistent excessive noise were identified through visual inspection and reconstructed using spherical spline interpolation. Continuous EEG data were band-pass filtered between 0.1 and 30 Hz and subsequently re-referenced to the average of all scalp electrodes. Ocular artifacts were corrected using independent component analysis (ICA) with the FastICA algorithm as implemented in MNE-Python. To improve ICA decomposition while retaining slow ERP activity in the data used for analysis, ICA weights were estimated from a copy of the continuous recording filtered between 1 and 30 Hz and were then applied to the 0.1–30 Hz filtered data. Components associated with blinks or eye movements were identified by inspecting their scalp distributions and temporal profiles and were removed from the continuous EEG signal before epoch extraction. Epochs were extracted separately for the adaptor face (S1) and the test face (S2), extending from 100 ms before to 500 ms after the onset of the corresponding stimulus. Each epoch was baseline-corrected using the 100-ms interval immediately preceding stimulus onset. Trials containing ethnicity-change targets were omitted from ERP analyses. Artifact rejection was implemented in two successive stages. First, before ICA correction, trials containing prominent blink activity during stimulus processing were identified because a blink occurring during face presentation could compromise perceptual encoding of the adaptor or test stimulus (Zhang et al., 2024). For this purpose, a moving-window peak-to-peak procedure was applied to Fp1 and Fp2 over the interval from −100 to 400 ms relative to stimulus onset, using a 200-ms window advanced in 10-ms steps. Trials were rejected when the peak-to-peak amplitude exceeded 100 μV. Second, after ICA correction, residual non-ocular artifacts were identified over the entire analysis epoch from −100 to 500 ms using the same moving-window procedure. Epochs were excluded when the peak-to-peak amplitude exceeded 80 μV in more than 20% of the scalp electrodes. Participants were included in the ERP analyses only if they contributed at least 20 artifact-free trials to each critical experimental condition.

### 2.5. Data Analyses

#### 2.5.1. Behavioral Data Analysis

A response was classified as a hit when participants pressed the response key on a trial in which the ethnicity of the presented face pair differed from that of the immediately preceding trial, and as a false alarm when a response occurred on a non-target trial. Reaction times were computed for correctly detected target trials only. Signal-detection sensitivity (d′) was calculated as z(hit rate) − z(false-alarm rate). To avoid infinite values when hit or false-alarm rates were at ceiling or floor, a log-linear correction was applied: corrected hit rate = (hits + 0.5)/(number of target trials + 1), and corrected false-alarm rate = (false alarms + 0.5)/(number of non-target trials + 1). Participants were excluded from ERP analyses if their d′ scores were more than three standard deviations below the mean of the EEG sample or if their target hit rate indicated failure to engage with the orthogonal task. Behavioral performance in the retained sample was summarized using descriptive statistics.

#### 2.5.2. ERP Data Analysis

ERP analyses focused on the P1, N170, P2, and N250 components. Electrode sites were selected from posterior scalp locations at which each component was most clearly expressed in the grand-average waveform collapsed across the Adapt and Nonadapt conditions. These electrode sites were also consistent with those commonly used to quantify face-related ERP components in previous studies (Rossion & Jacques, 2008; H. Tanaka & Jiang, 2024). P1 and P2 were measured at O1 and O2, whereas N170 and N250 were measured at P7 and P8.

For each component, peak latency was identified from the grand-average waveform collapsed across S1 and S2 and across the Adapt and Nonadapt conditions. Collapsing across conditions allowed the measurement windows to be defined independently of the experimental contrast, consistent with the collapsed-localizer approach (Luck & Gaspelin, 2017). Mean amplitude was then calculated within a fixed 40-ms interval centred on the identified peak. This width was selected to provide a stable estimate of activity around the component maximum while limiting temporal overlap between adjacent components, particularly the P2 and N250; comparable 40-ms peak-centred windows have been used in previous visual and face-ERP studies (Jin et al., 2023; Pegado et al., 2014). The resulting measurement windows were 94–134 ms for the P1, 143–183 ms for the N170, 200–240 ms for the P2, and 253–293 ms for the N250. The same electrode sites and fixed measurement windows were applied to all participants, both Pair Type conditions, and the corresponding S1 control analyses. Mean amplitudes were extracted separately for the left- and right-hemisphere electrodes.

For each component, S2 mean amplitudes were submitted to a repeated-measures ANOVA with Pair Type (Adapt, Nonadapt) and Hemisphere (left, right) as within-participant factors. S1-locked ERPs served as a control analysis to verify that any S2 effects were not attributable to pre-existing differences between the Adapt and Nonadapt conditions introduced at the adaptor stage. All tests were two-tailed, with significance evaluated at *p* < 0.05. When a significant Pair Type × Hemisphere interaction was observed, simple-effects tests examined the effect of Pair Type separately at the left and right hemisphere sites. Because these analyses involved two planned hemisphere-specific comparisons, significance was evaluated using a Bonferroni-adjusted alpha level of 0.025.

To directly assess changes in ERP amplitude from S1 to S2, mean amplitudes for each component were entered into repeated-measures ANOVAs with Stimulus Position (S1, S2), Pair Type (Adapt, Nonadapt), and Hemisphere (left, right) as within-participant factors. The critical Stimulus Position × Pair Type interaction tested whether the S1-to-S2 amplitude change differed between repeated- and changed-spacing trials. Significant interactions involving hemisphere were followed by separate analyses for the left and right hemispheres. Direct S1-versus-S2 comparisons within each Pair Type condition were conducted using paired-samples t tests, with significance evaluated using a Bonferroni-adjusted alpha level of 0.025.

Partial eta squared ($\eta_{p}^{2}$) was reported for ANOVA effects. All statistical analyses were performed using JASP 0.97.0.

#### 2.5.3. Cluster-Based Permutation Analysis

As a complementary analysis, nonparametric cluster-based permutation tests were used to characterize the spatiotemporal distribution of the Pair Type effect without restricting inference to predefined ERP windows or electrode regions (Maris & Oostenveld, 2007). For each participant, difference waveforms were computed as Nonadapt minus Adapt separately for S2 and S1. One-sample cluster-based permutation tests examined whether these different waveforms differed from zero across all scalp electrodes over the 0–500 ms post-stimulus interval. Spatiotemporally adjacent samples exceeding a two-tailed cluster-forming threshold of *p* < 0.05 were grouped into clusters, and cluster statistics were defined as the sum of t-values within each cluster. Cluster significance was evaluated using 5000 sign-flipping permutations, with family-wise error controlled at the cluster level at α = 0.05. Because cluster-based inference does not provide precise estimates of effect onset, duration, or localization, these analyses were interpreted as complementary evidence for spatially and temporally extended condition effects.

## 3. Results

### 3.1. Behavioral Results

In the final sample, participants detected ethnicity-change targets with a mean hit rate of 94.18% (*SD* = 14.14%) and a mean false-alarm rate of 2.97% (*SD* = 3.32%). Mean corrected sensitivity was d′ = 3.51 (*SD* = 0.68), and mean reaction time for correctly detected targets was 608 ms (*SD* = 180 ms). These results indicate that the retained participants were sufficiently engaged with the orthogonal task during EEG recording.

### 3.2. ERP Results

#### 3.2.1. S1-Locked Control Analyses

For S1-locked P1, N170, and P2 (see Figure S1), no significant Pair Type main effects or Pair Type × Hemisphere interactions were observed (all *F*s(1, 69) < 3.20, all *p*s ≥ 0.078, all $\eta_{p}^{2}$s ≤ 0.044). Significant hemisphere main effects were present for P1, *F*(1, 69) = 4.23, *p* = 0.043, $\eta_{p}^{2}$ = 0.058, and P2, *F*(1, 69) = 6.44, *p* = 0.013, $\eta_{p}^{2}$ = 0.085, reflecting larger amplitudes over left than right hemisphere sites; these lateralization effects were unrelated to the experimental manipulation.

For S1-locked N250, no significant main effect of Pair Type was found, *F*(1, 69) = 0.84, *p* = 0.362, $\eta_{p}^{2}$ = 0.012, nor a significant hemisphere main effect, *F*(1, 69) = 1.13, *p* = 0.292, $\eta_{p}^{2}$ = 0.016. However, the Pair Type × Hemisphere interaction was significant, *F*(1, 69) = 9.04, *p* = 0.004, $\eta_{p}^{2}$ = 0.116. Simple-effects tests, evaluated using a Bonferroni-adjusted alpha level of 0.025, showed no Pair Type effect at P7, *F*(1, 69) = 0.54, *p* = 0.467, but a significant effect at P8, *F*(1, 69) = 5.44, *p* = 0.023. At P8, Adapt trials elicited a more negative-going response (*M* = −0.16 μV, *SD* = 4.28) than Nonadapt trials (*M* = 0.24 μV, *SD* = 4.27). Importantly, this right-posterior pattern was opposite in direction to the N250 condition effect observed at S2.

#### 3.2.2. S2-Locked ERPs

**Figure 2:**
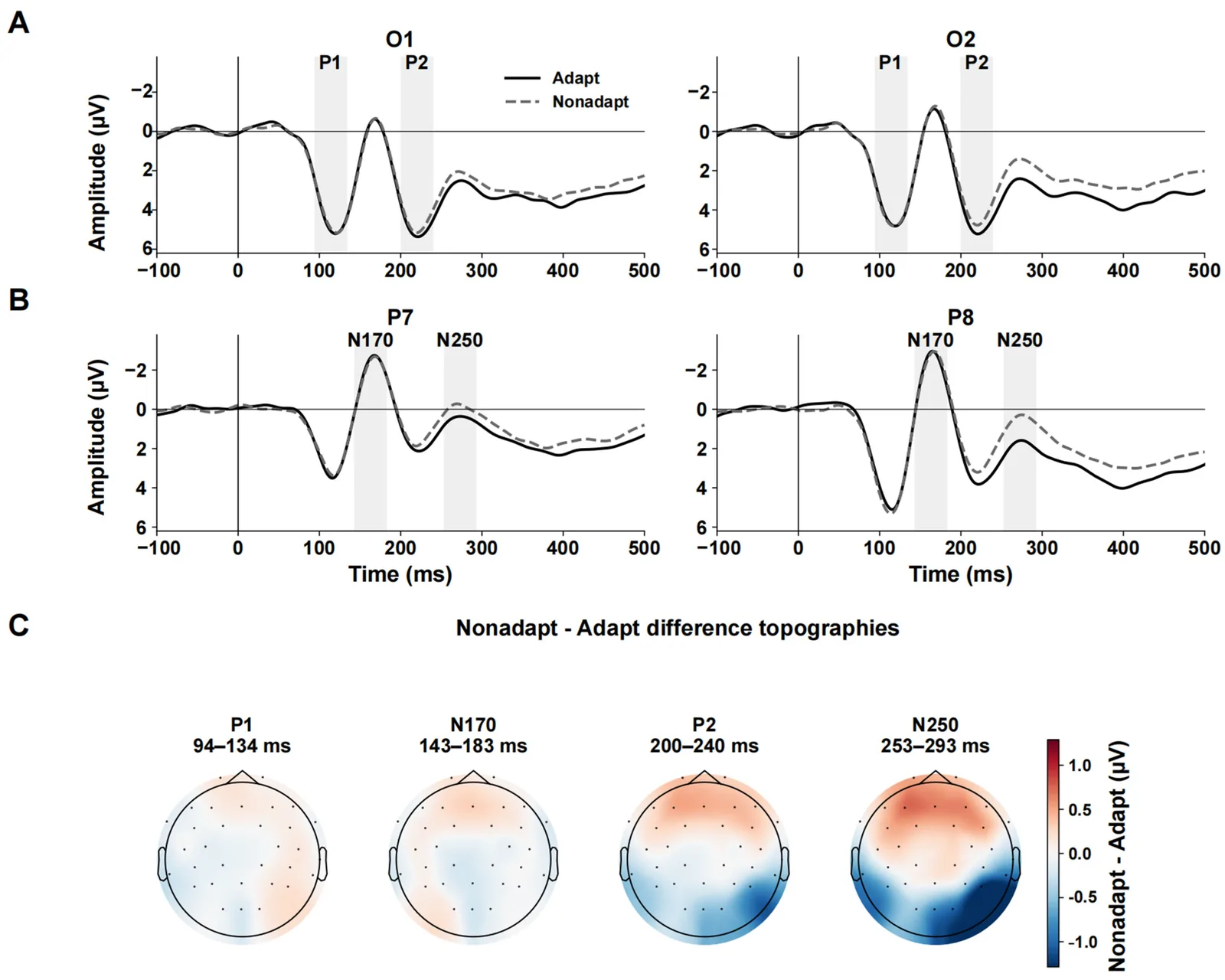
Grand-averaged ERP waveforms and Nonadapt-Adapt difference topographies for S2-locked responses. (**A**) Grand-averaged ERP waveforms recorded at occipital electrodes O1 and O2. Solid lines indicate the Adapt condition; dashed lines indicate the Nonadapt condition. Shaded rectangles denote the mean-amplitude analysis windows for the P1 (94–134 ms) and P2 (200–240 ms) components. (**B**) Grand-averaged ERP waveforms recorded at posterior temporal electrodes P7 and P8. Shaded rectangles denote the analysis windows for the N170 (143–183 ms) and N250 (253–293 ms) components. Negative amplitude is plotted upward. (**C**) Scalp topographies of the Nonadapt-Adapt difference (in μV) averaged within each analysis time window. Warm colors (red) indicate greater positivity for Nonadapt relative to Adapt; cool colors (blue) indicate greater positivity for Adapt relative to Nonadapt.

**P1 and N170.** Neither component showed reliable modulation by second-order relational continuity (see Figure 2). For P1, neither the Pair Type main effect, *F*(1, 69) = 0.01, *p* = 0.942, $\eta_{p}^{2}$ < 0.001, nor the Pair Type × Hemisphere interaction, *F*(1, 69) = 0.48, *p* = 0.492, $\eta_{p}^{2}$ = 0.007, was significant. Similarly, N170 showed no significant Pair Type effect, *F*(1, 69) = 0.04, *p* = 0.848, $\eta_{p}^{2}$ = 0.001, or Pair Type × Hemisphere interaction, *F*(1, 69) = 0.36, *p* = 0.551, $\eta_{p}^{2}$ = 0.005.

**P2.** A significant Pair Type main effect was observed, *F*(1, 69) = 6.02, *p* = 0.017, $\eta_{p}^{2}$ = 0.080. Adapt trials elicited larger positive P2 amplitudes than Nonadapt trials across both hemispheres (left: *M* = 4.92, *SD* = 3.47 μV vs. *M* = 4.64, *SD* = 3.54 μV; right: *M* = 4.75, *SD* = 3.61 μV vs. *M* = 4.24, *SD* = 3.43 μV), reflecting a significant condition difference in the P2 time window (see Figure 2A). Neither the hemisphere main effect, *F*(1, 69) = 1.64, *p* = 0.204, $\eta_{p}^{2}$ = 0.023, nor the Pair Type × Hemisphere interaction, *F*(1, 69) = 2.63, *p* = 0.109, $\eta_{p}^{2}$ = 0.037, reached significance.

**N250.** A robust Pair Type main effect was found, *F*(1, 69) = 46.57, *p* < 0.001, $\eta_{p}^{2}$ = 0.403, and was qualified by a significant Pair Type × Hemisphere interaction, *F*(1, 69) = 9.27, *p* = 0.003, $\eta_{p}^{2}$ = 0.118. A significant hemisphere main effect was also present, *F*(1, 69) = 7.93, *p* = 0.006, $\eta_{p}^{2}$ = 0.103. Simple-effects tests, evaluated using a Bonferroni-adjusted alpha level of 0.025, revealed significant Pair Type effects at both P7, *F*(1, 69) = 13.27, *p* < 0.001, and P8, *F*(1, 69) = 41.57, *p* < 0.001. Nonadapt trials elicited more negative N250 amplitudes than Adapt trials across both hemispheres (left: *M* = −0.08, *SD* = 2.72 μV vs. *M* = 0.49, *SD* = 2.77 μV; right: *M* = 0.54, *SD* = 3.63 μV vs. *M* = 1.82, *SD* = 3.45 μV, see Figure 2B).

In addition, following FDR correction across all S2 Pair Type effects and interactions, the main effects for P2 and N250 and the Pair Type × Hemisphere interaction for N250 remained significant. All other effects did not reach significance after correction.

#### 3.2.3. Direct Comparisons of S1- and S2-Locked Responses

For the P1, the Stimulus Position × Pair Type interaction was not significant, *F*(1, 69) = 0.06, *p* = 0.804, $\eta_{p}^{2}$ = 0.001, nor was the Stimulus Position × Pair Type × Hemisphere interaction, *F*(1, 69) = 0.09, *p* = 0.765, $\eta_{p}^{2}$ = 0.001. P1 amplitude increased from S1 to S2 in both the Adapt and Nonadapt conditions, as indicated by a significant main effect of Stimulus Position, *F*(1, 69) = 6.86, *p* = 0.011, $\eta_{p}^{2}$ = 0.090. Similarly, for the N170, neither the Stimulus Position × Pair Type interaction, *F*(1, 69) = 0.34, *p* = 0.561, $\eta_{p}^{2}$ = 0.005, nor the three-way interaction with Hemisphere, *F*(1, 69) = 2.13, *p* = 0.149, $\eta_{p}^{2}$ = 0.030, was significant. The N170 became less negative from S1 to S2 in both Pair Type conditions, as indicated by a significant main effect of Stimulus Position, *F*(1, 69) = 61.63, *p* < 0.001, $\eta_{p}^{2}$ = 0.472.

For the P2, the Stimulus Position × Pair Type interaction was significant, *F*(1, 69) = 12.63, *p* < 0.001, $\eta_{p}^{2}$ = 0.155, and was not further qualified by Hemisphere, *F*(1, 69) = 2.89, *p* = 0.094, $\eta_{p}^{2}$ = 0.040. The P2 showed a numerical increase from S1 to S2 in the Adapt condition, *t*(69) = 2.08, *p* = 0.041, but this comparison did not meet the Bonferroni-adjusted significance criterion of 0.025. No corresponding S1-to-S2 change was observed in the Nonadapt condition, *t*(69) = −0.07, *p* = 0.944.

For the N250, the Stimulus Position × Pair Type interaction was significant, *F*(1, 69) = 26.62, *p* < 0.001, $\eta_{p}^{2}$ = 0.278, and was qualified by a significant Stimulus Position × Pair Type × Hemisphere interaction, *F*(1, 69) = 16.53, *p* < 0.001, $\eta_{p}^{2}$ = 0.193. Separate analyses showed a robust Stimulus Position × Pair Type interaction over the right hemisphere, *F*(1, 69) = 39.44, *p* < 0.001, $\eta_{p}^{2}$ = 0.364, but not over the left hemisphere, *F*(1, 69) = 3.32, *p* = 0.073, $\eta_{p}^{2}$ = 0.046. Over the right hemisphere, the N250 became less negative from S1 to S2 in the Adapt condition (see Figure S1), *t*(69) = 4.34, *p* < 0.001, meeting the Bonferroni-adjusted significance criterion. No reliable S1-to-S2 change was observed in the Nonadapt condition, *t*(69) = 0.74, *p* = 0.465.

#### 3.2.4. Cluster-Based Permutation Analyses

To complement the mean-amplitude ANOVAs and examine adaptation effects without a priori time-window assumptions, cluster-based permutation tests were conducted across all electrodes and time points (0–500 ms) for both S1- and S2-locked epochs.

For S1-locked responses, no significant spatiotemporal clusters were identified (all cluster *p*s > 0.13), providing data-driven confirmation that the Adapt and Nonadapt conditions did not systematically differ at the adaptor stage.

**Figure 3:**
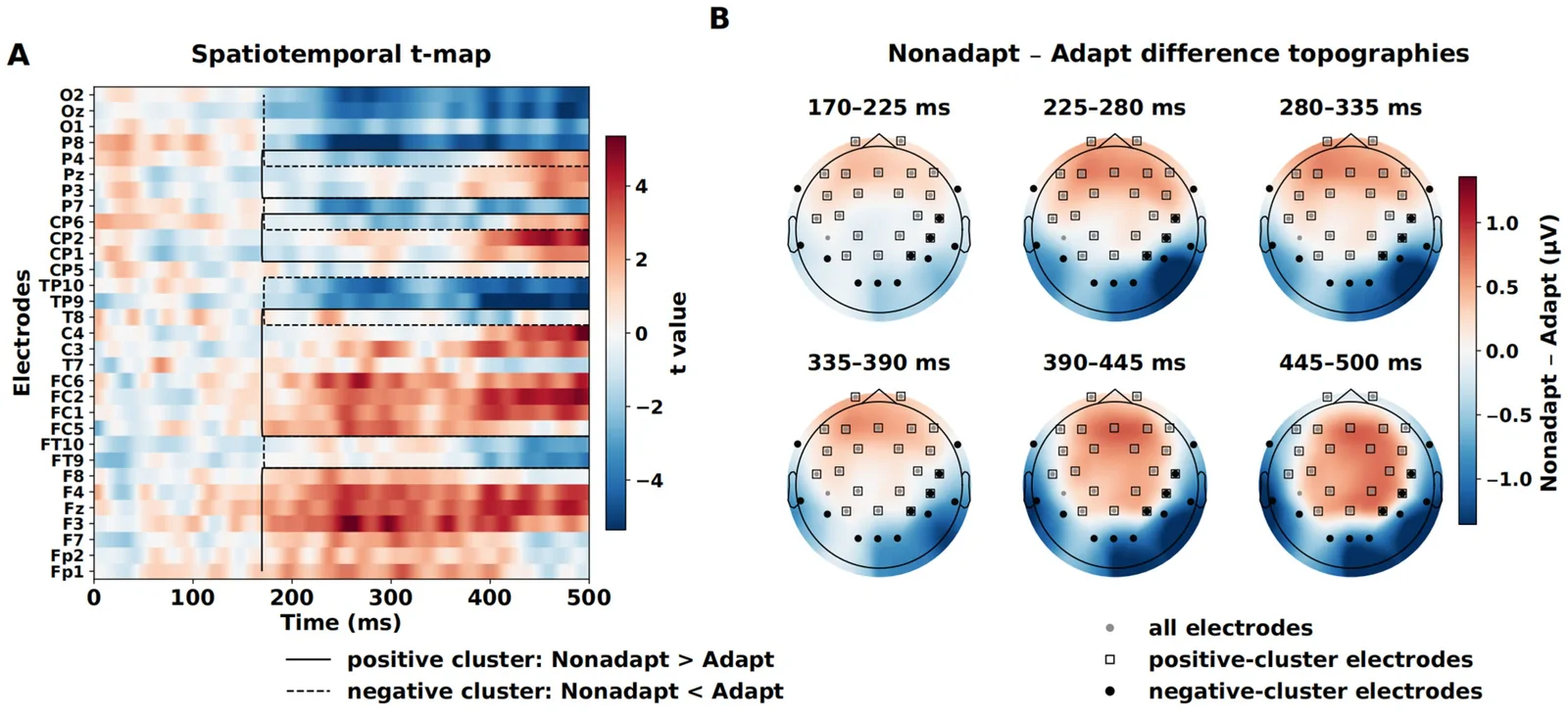
Cluster-based permutation results for S2 adaptation effects. (**A**) Spatiotemporal t-map for the S2-locked Nonadapt-minus-Adapt contrast across all scalp electrodes from 0 to 500 ms. Solid black contours indicate the significant positive cluster, reflecting larger amplitudes for Nonadapt than Adapt trials. Dashed black contours indicate the significant negative cluster, reflecting smaller amplitudes for Nonadapt than Adapt trials. (**B**) Scalp topographies of the Nonadapt-minus-Adapt difference averaged across consecutive 55-ms windows from 170 to 500 ms. Open squares indicate electrodes belonging to the positive cluster, and filled circles indicate electrodes belonging to the negative cluster.

Cluster-based permutation tests on S2-locked ERPs (0–500 ms) identified two significant spatiotemporal clusters (both *p*s = 0.0002, see Figure 3). A negative cluster (Nonadapt < Adapt) emerged at 172 ms and persisted through 500 ms, encompassing 12 posterior and temporal electrodes with a right-hemisphere predominance (FT9, TP9, P7, O1, Oz, O2, P4, P8, TP10, CP6, T8, FT10; peak *t* = −6.44 at 275 ms, electrode P8). A positive cluster (Nonadapt > Adapt) emerged at 170 ms and extended through 500 ms across 21 frontal, central, and parietal electrodes (Fp1, Fz, F3, F7, FC5, FC1, C3, T7, CP1, Pz, P3, P4, CP6, CP2, C4, T8, FC6, FC2, F4, F8, Fp2; peak *t* = 6.01 at 499 ms, electrode C4). The posterior negative cluster overlapped with the P2 and N250 time ranges identified in the component-based analyses, whereas the broader positive cluster was not treated as a direct index of second-order relational encoding.

## 4. Discussion

The present study used a rapid-adaptation paradigm to characterise the temporal progression of neural sensitivity to repeated versus changed second-order facial relations across the P1, N170, P2, and N250 time ranges. By using adaptor-test pairs derived from the same source identity while varying whether the metric spacing among internal facial features was repeated or changed, the design allowed us to assess ERP sensitivity to relational continuity and change within same-source face pairs. The P1 and N170 showed no reliable Adapt-Nonadapt modulation, whereas a sustained posterior condition difference emerged during the post-N170 interval spanning the conventionally defined P2 and N250 time windows.

### 4.1. No Reliable Second-Order Relational Modulation at Early ERP Stages

The absence of reliable Adapt–Nonadapt modulation at the P1 and N170 indicates that repeated versus changed inter-feature spacing was not differentiated at these early ERP stages. This finding is consistent with the view that the N170 primarily reflects early face-sensitive structural encoding, such as the detection or integration of a canonical face structure, rather than the reliable encoding of fine-grained metric distances that individuate one face from another (Rossion, 2013; Schweinberger & Neumann, 2016). Consistent with this view, second-order spacing manipulations that leave the canonical first-order face structure intact have often failed to modulate the N170 in standard ERP paradigms (Halit et al., 2000; Mercure et al., 2008). The present results extend this pattern to a rapid-adaptation context using unfamiliar faces, providing evidence that such null effects generalize beyond passive-viewing and direct comparison paradigms. These null findings are also consistent with evidence that varying face identity strength through morphing modulates later P2 and N250 responses while leaving P1 and N170 largely unaffected (Zheng et al., 2012). The direct S1–S2 comparisons further qualified this early-stage pattern. Both the P1 and N170 changed reliably from the first to the second face, with a larger P1 and a less negative N170 response to the second face. However, the magnitude of these changes did not differ reliably depending on whether internal-feature spacing was repeated or changed. Thus, the early components showed general changes across stimulus position, but did not clearly differentiate relational repetition from change under the present conditions.

It is nonetheless important to avoid the stronger claim that early processing stages are entirely insensitive to second-order relational information. As reviewed in the Introduction, N170 sensitivity to second-order relational changes has been reported under specific conditions, including prior face familiarization, repetition/adaptation paradigms involving expanded or contracted inner features, and long-duration adaptation to altered second-order configurations (Scott & Nelson, 2006; Vakli et al., 2014; Zimmer & Kovács, 2011). The discrepancy between the present N170 null effect and the long-term adaptation effect reported by Vakli et al. (2014) may partly reflect differences in adaptation duration: their study used 5-s adaptation to normal or vertically stretched faces, whereas the present study used brief within-trial adaptation. This possibility is consistent with evidence that N170 adaptation effects are influenced by adaptor duration and interstimulus interval (e.g., Feuerriegel et al., 2015). Future studies could directly manipulate adaptation duration to test whether stronger or longer exposure to altered second-order configurations is enough to elicit N170 adaptation effects. Thus, the present findings do not imply that second-order relational information can never influence the N170; rather, they suggest that such sensitivity is not reliably expressed under the current rapid-adaptation conditions with unfamiliar faces.

### 4.2. Second-Order Relational Sensitivity Beyond the N170: P2 and N250

In contrast to the absence of reliable P1 and N170 effects, the P2 showed a bilateral repetition-enhancement effect, with larger positive amplitudes when the inter-feature spacing of the test face matched that of the adaptor. This finding is consistent with previous evidence linking the P2/P200 to the processing of facial spatial configuration, prototypicality, and distance-to-norm in face space (Schweinberger & Neumann, 2016; Wuttke & Schweinberger, 2019). Importantly, the present study did not directly manipulate facial prototypicality. Instead, it manipulated whether the same metric relations among internal features were repeated or changed across two successive presentations of the same identity. The P2 effect may therefore reflect a short-term relational match between the adaptor and test face: when the inter-feature spacing was repeated, the test face matched the relational template established by the adaptor, yielding an enhanced P2 response. This repetition-enhancement pattern should not be taken as inconsistent with an adaptation framework, because repetition effects can be expressed as either enhancement or suppression depending on the component, stimulus property, and processing context (De Gardelle et al., 2013; Segaert et al., 2013). Thus, the P2 finding suggests that repeated second-order relational structure facilitated a mid-latency relational-match or template-based comparison process after the N170.

The N250 provided the clearest evidence that changing inter-feature spacing within the same facial identity modulated a later stage of face processing associated with identity-related representations. Nonadapt trials elicited larger posterior negativities than Adapt trials, indicating that a change in spacing configuration produced a release from adaptation in the N250 time window. This effect should be distinguished from the classic N250r repetition-priming effect, in which repeated familiar faces elicit enhanced posterior negativity relative to unrepeated faces (Schweinberger et al., 2002; Schweinberger & Neumann, 2016). In the present design, facial identity was repeated in both Adapt and Nonadapt trials; the critical manipulation was whether the internal spacing configuration was preserved or changed. Thus, the larger N250 negativity for Nonadapt trials is most directly interpretable as sensitivity to relational mismatch within the same source identity. Because S1 is assumed to establish a short-lived representation of the face’s relational configuration, a spacing change at S2 creates a discrepancy between the incoming relational information and the currently active face representation. Resolving this discrepancy may require greater representational updating, thereby contributing to the larger N250 negativity observed in the Nonadapt condition. This interpretation is consistent with evidence linking the N250 to the activation of familiar or newly acquired individual-face representations (Schweinberger & Neumann, 2016; J. W. Tanaka et al., 2006). When the spacing configuration was repeated, the currently active identity-related representation may have required less relational updating, consistent with the attenuated N250 response observed in the Adapt condition. While ERP scalp distributions cannot directly resolve cortical generators, the right-weighted posterior distribution of the present N250 effect is broadly consistent with prior work showing that N250/N250r repetition effects are maximal over right temporal sites and may involve fusiform sources (Schweinberger et al., 2004; Kaufmann et al., 2009), as well as with evidence implicating right occipito-temporal cortex in the representation of individual faces (Jacques & Rossion, 2009; Lochy et al., 2020).

Although the condition effect reached significance in both the conventionally defined P2 and N250 windows, the difference waveforms and scalp topographies suggest that these findings should not necessarily be interpreted as two distinct effects operating in opposite directions. In the Nonadapt-minus-Adapt contrast, both time windows were characterised by a posterior negative-going difference, which formed part of a sustained condition effect extending across the post-N170 interval. The P2- and N250-window results may therefore reflect different measurements of the same sustained voltage shift rather than independent relational-matching and representational-updating mechanisms. The overlap with the P2 and N250 time ranges remains broadly compatible with previous accounts linking these intervals to spatial-relational evaluation and identity-related processing, respectively, but the present data do not allow these functional contributions to be separated.

The direct S1–S2 comparisons further clarified the response trajectories underlying the later S2 condition effects. For the P2, the change from the first to the second face differed depending on whether internal-feature spacing was repeated or changed. The P2 showed a numerical increase when spacing was repeated but remained largely unchanged when spacing was altered; however, the increase within the repeated-spacing condition did not meet the Bonferroni-adjusted significance criterion. The interaction across stimulus position and spacing condition, rather than the individual within-condition contrast, therefore provides the primary evidence for condition-dependent P2 modulation. For the N250, the differential response trajectory was most pronounced over the right hemisphere. The response became less negative from the first to the second face when spacing was repeated, whereas no reliable change occurred when spacing was altered. Because a smaller condition difference in the opposite direction was already present at the first face, this finding is more appropriately interpreted as a condition-dependent S1-to-S2 trajectory than as an unqualified measure of pure adaptation. Together, these direct comparisons complement the primary S2 analyses by showing that relational repetition and change were associated with different later response trajectories under the present conditions.

In addition, the cluster-based permutation analysis provided a data-driven complement to the component-based analyses. Significant Adapt–Nonadapt differences emerged primarily over posterior electrodes during a sustained post-N170 interval spanning the conventionally defined P2 and N250 time ranges, whereas no reliable clusters were detected during the P1 or N170 intervals. The posterior cluster began at approximately 172 ms and persisted through the end of the analysed epoch, encompassing both the P2 and N250 measurement windows. Its sustained negative polarity and broadly similar posterior distribution across this interval suggest that the component-based P2- and N250-window findings may reflect different measurements of a temporally extended post-N170 condition difference, rather than a clear transition between two separable mechanisms. The timing of this sustained effect overlaps intervals previously associated with spatial-relational evaluation and identity-related face processing (Burkhardt et al., 2010; Kaufmann et al., 2009; Kaufmann & Schweinberger, 2012; J. W. Tanaka et al., 2006; Schweinberger & Neumann, 2016), but the present data do not permit these functional contributions to be independently isolated. Importantly, the permutation analysis showed that the condition differences extended across contiguous time points and neighbouring posterior electrodes rather than being restricted to the predefined component windows or selected electrodes. It therefore provides converging evidence for a sustained posterior post-N170 response that differentiated repeated from changed second-order facial relations. However, the absence of significant early clusters should not be interpreted as evidence that second-order information was entirely absent from P1 or N170 processing.

### 4.3. Limitations and Future Directions

Several limitations of the present study warrant acknowledgment. First, the stimulus set consisted of unfamiliar faces, and the second-order manipulation was restricted to the horizontal and vertical positions of the eyes and mouth. Although these features are central to second-order relational processing, other forms of metric variation, such as nose width, brow height, facial aspect ratio, or spacing among additional internal features, may engage partially different stages of face processing or show different lateralization patterns. Future studies could therefore test whether the present P2 and N250 effects generalize across different types of metric facial variation. It would also be informative to compare second-order spacing changes with featural changes within the same adaptation design. Such a comparison would help determine whether the observed P2 and N250 effects are specific to relational information or instead reflect a broader sensitivity to within-identity facial change.

Second, the present rapid-adaptation paradigm used brief adaptor presentations and a relatively short adaptor-test interval. This design was well suited to examining rapid sensitivity to relational repetition or change within the same facial identity, but it may have limited the depth of adaptation at earlier processing stages. Previous work suggests that N170 adaptation effects can depend on adaptor duration and interstimulus interval, making these parameters important for interpreting null effects in the N170 range (Feuerriegel et al., 2015). Future studies should systematically vary adaptor duration and SOA to determine whether the absence of reliable N170 modulation holds across a wider range of adaptation conditions.

A further limitation is that perceived identity continuity across the manipulated face images was not independently verified. Although each pair was derived from the same photographed individual and retained the same local features and first-order facial layout, changes in internal-feature spacing may have influenced perceived identity similarity. The present findings should therefore be interpreted as responses to repeated versus changed spacing within same-source face pairs, rather than as evidence concerning relational variation within a perceptually invariant identity. Future studies could include an independent behavioural assessment of perceived identity continuity, such as identity-similarity ratings or same/different identity judgments.

## 5. Conclusions

The present study shows that, under rapid-adaptation conditions, repeated versus changed second-order facial relations were most reliably differentiated during a sustained post-N170 interval spanning the conventionally defined P2 and N250 time ranges. The P1 and N170 did not reliably distinguish repeated from changed spacing, whereas repeated spacing elicited a more positive-going posterior response than changed spacing during the later interval. Although this effect overlapped both the P2 and N250 windows, the present findings do not establish that these windows reflect two separable mechanisms. Rather, they identify a sustained post-N170 period in which sensitivity to second-order relational continuity and change was most clearly expressed under the present conditions.

## Data Availability

Data supporting the results of this study are available from the corresponding author upon reasonable request.

## Supplementary Materials

The following supporting information can be downloaded at: https://www.mdpi.com/article/10.3390/bs16081301/s1, Figure S1: S1- and S2-locked ERP waveforms in the Adapt and Nonadapt conditions; Supplementary Analysis S1: Exploratory associations between ERP adaptation effects and face-memory performance.
