# Supplementary material for: Temporal Dynamics of Sensitivity to Second-Order Facial Relations: Evidence from a Rapid ERP Adaptation Paradigm
Source: Behav Sci (Basel). 2026 Jul 31;16(8):1301. doi: 10.3390/bs16081301 (PMC13509208; doi:10.3390/bs16081301)
Supplement: Supplementary file 1 [file behavsci-16-01301-s001.zip › Supplementary Analysis S1.pdf]

Exploratory associations between ERP adaptation effects and face-memory performance

Rationale and measure

Following the EEG session, participants completed the Cambridge Face Memory Test–Chinese version (CFMT-Chinese; McKone et al., 2017), an adaptation of the original Cambridge Face Memory Test using Chinese-ethnic face stimuli (Duchaine & Nakayama, 2006). The task uses a three-alternative forced-choice format and has a maximum score of 72. One participant had incomplete CFMT-Chinese data and was excluded from these exploratory analyses, leaving a sample of 69 participants.

These analyses explored whether ERP sensitivity to repeated versus changed second-order facial relations was associated with broader face-memory ability. They were considered exploratory because the CFMT-Chinese measures face learning and recognition across multiple trials and does not directly assess sensitivity to internal-feature spacing.

Analysis

Because reliable S2 Pair Type effects were observed for the P2 and N250, condition-effect indices were calculated for these components. The bilateral P2 repetition-enhancement index was calculated as Adapt minus Nonadapt mean amplitude, such that larger positive values indicated a larger P2 response to repeated spacing. The bilateral N250 condition-effect index was calculated as Nonadapt minus Adapt mean amplitude; because Nonadapt trials elicited more negative-going responses, more negative values indicated a larger N250 condition effect. Given the Pair Type × Hemisphere interaction observed for the N250, left- and right-hemisphere N250 indices were also examined exploratorily. Pearson correlations were calculated between each ERP index and CFMT-Chinese accuracy. The four correlation *p*-values were corrected together using the false-discovery-rate procedure.

Results

Mean accuracy on the CFMT-Chinese was .78 (*SD* = .13). Because reliable S2 Pair Type effects were observed for P2 and N250, follow-up analyses examined whether the magnitude of these later ERP modulations was related to face recognition ability. The P2 condition-effect index was calculated as Adapt minus Nonadapt mean amplitude, such that larger positive values reflected greater repetition-related enhancement. The N250 condition-effect index was calculated as Nonadapt minus Adapt mean amplitude; because Nonadapt trials elicited more negative-going responses than Adapt trials, more negative values reflected a larger N250 adaptation-suppression effect. Hemisphere-specific N250 correlations were examined as exploratory analyses. All p-values were corrected using the FDR procedure across the four comparisons.

None of the four correlations survived FDR correction. The bilateral P2 repetition-enhancement index showed a small positive association with CFMT-Chinese accuracy that did not survive correction (r = .261, p = .030, pFDR = .120). The bilateral N250 adaptation index was not associated with CFMT-Chinese accuracy (r = −.061, p = .617, pFDR = .689), nor were the exploratory left-hemisphere (r = .049, p = .689, pFDR = .689) or right-hemisphere (r = −.123, p = .316, pFDR = .632) N250 effects. Thus, the

magnitude of the observed P2 and N250 adaptation effects was not reliably associated with objective face recognition performance in the present sample.

Discussion

No Reliable Associations Between ERP Relational Sensitivity and Face Recognition Ability

The exploratory correlation analyses revealed no reliable associations between the P2 or N250 Adapt–Nonadapt effects and CFMT-Chinese performance. This null result does not rule out a role for second-order relational processing in face recognition ability. Rather, it suggests that, in the present sample and task context, trial-level neural sensitivity to repeated versus changed inter-feature spacing did not reliably predict individual differences in broader face memory performance. One plausible explanation is that the ERP adaptation effects and CFMT-Chinese performance reflect partly non-overlapping aspects of face processing. The present ERP paradigm measured an incidental, short-term response to relational repetition or change within the same facial identity, whereas the CFMT-Chinese requires learning unfamiliar faces and recognizing them across multiple test trials, thereby engaging broader encoding, retention, retrieval, and decision processes (Duchaine & Nakayama, 2006; McKone et al., 2017). Neural sensitivity to second-order relational change may therefore be only one component of the broader set of processes that support behavioral face recognition. In addition, the relatively homogeneous university sample may have restricted the range of face memory ability, limiting sensitivity to individual-difference associations. Future studies using larger and more ability-diverse samples, together with behavioral measures that more directly target second-order relational sensitivity, would provide a stronger test of the relationship between ERP adaptation effects and face recognition ability.
